# Implementation of intelligent All‐in‐one technology in rectal cancer radiotherapy: A retrospective study on automation efficiency and safety

**DOI:** 10.1002/acm2.70236

**Published:** 2025-09-10

**Authors:** Haoyang Zhai, Jiazhou Wang, Weigang Hu

**Affiliations:** ^1^ Department of Radiation Oncology, Fudan University Shanghai Cancer Center Department of Oncology Shanghai Clinical Research Center for Radiation Oncology Shanghai Key Laboratory of Radiation Oncology Shanghai Medical College, Fudan University Shanghai China

**Keywords:** All‐in‐one, automated planning, clinical practice, dosimetric metric

## Abstract

**Purpose:**

This study aims to assess percentage of automated AIO plans that met clinical treatment standards of radiotherapy plans generated by the fully automated All‐in‐one (AIO) process.

**Methods:**

The study involved 117 rectal cancer patients who underwent AIO treatment. Fully automated regions of interest (ROI) and treatment plans were developed without manual intervention, comparing them to manually generated plans used in clinical practice. Geometric and dosimetric metrics were collected from both automated and manual plans. The relationship between the geometric and dosimetric metrics of the planning target volume (PTV) was evaluated using Spearman correlation analysis. The interquartile range (IQR) method was applied to determine the percentage of automated plans meeting clinical requirements. Additionally, dosimetric metrics for organs at risk (OAR) were compared between automated and manual plans using paired *t*‐tests. The reasons for dose discrepancies were examined based on target volume.

**Results:**

Spearman correlation analysis showed a moderate correlation between geometric metrics and the conformity index (△CI) in dosimetric metrics. The correlation coefficients were as follows: Hausdorff distance (HD, |ρ| = 0.458, *p* < 0.01), Mean deviation area (MDA, |ρ| = 0.565, *p* < 0.01), Dice similarity coefficient (DSC, |ρ| = 0.631, *p* < 0.01), and Jaccard index (JI, |ρ| = 0.632, *p* < 0.01). Statistical analysis revealed that the mean doses to the bladder and bilateral femoral heads were significantly lower in automated plans compared to manual ones (*p* < 0.01). This difference is likely due to variations in ROI delineation between automated and manual methods. The IQR method showed that 81.2% of automated AIO plans met clinical requirements without manual intervention.

**Conclusion:**

In routine clinical practice, approximately 81.2% of automated AIO plans met clinical requirements without requiring manual intervention.

## 1 INTRODUCTION

The incidence and mortality rates of rectal cancer are rising globally, presenting a significant public health challenge. Global cancer statistics indicate that rectal cancer is now one of the most prevalent tumors worldwide.^1^ Radiotherapy (RT) plays a crucial role in treating rectal cancer. Traditional RT processes involve manual steps like target contouring and treatment planning, making them time‐consuming and prone to subjective bias.^2^ Manual treatment planning is highly dependent on the experience and expertise of radiation oncologists, which can introduce variability in treatment plans, particularly in complex cases where precise dose distribution and target delineation are critical.^3^, ^4^

**FIGURE 1:**
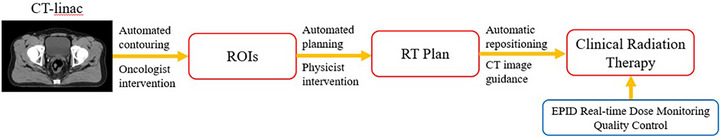
Workflow of All‐in‐one.

In response, AIO RT technology has emerged to enhance the efficiency and accuracy of treatment through automation. As illustrated in Figure 1, AIO technology integrates several steps—automated contouring, plan optimization, and dose calculation—into a single platform. This integration reduces manual intervention and increases efficiency.^5^, ^6^, ^7^ Notably, the entire AIO process can be completed in a brief timeframe. For example, a study achieved completion of the initial RT session in just 23 min, while traditional methods may take several days or even weeks.^8^ Research suggests that AIO technology generates clinically compliant treatment plans more quickly than traditional approaches and may lower the risk of treatment‐related complications.^9^ Additionally, the incorporation of artificial intelligence into treatment planning enhances accuracy and reduces variability.^10^, ^11^

Despite these potential benefits, manual planning remains the dominant approach in many clinics. This persistence is largely due to the skepticism among radiation oncologists regarding the reliability and safety of automated systems, particularly in complex cases where the accuracy of dose distribution and target delineation is critical. Many clinicians are concerned that automated plans may not adequately address the nuances of individual patient anatomy or the variability in tumor characteristics, leading to a preference for manual intervention to ensure treatment quality.^12^, ^13^ This skepticism highlights the need for systematic evaluation of AIO technology in clinical practice to demonstrate its reliability and safety.

This study aims to address these concerns by analyzing data from 117 patients with rectal tumors who underwent AIO treatment, focusing on the proportion of automated AIO plans that meet clinical requirements without manual intervention. By providing evidence on the reliability and safety of AIO technology, this study seeks to facilitate its broader acceptance and application in clinical practice.

## 2 MATERIALS AND METHODS

**FIGURE 2:**
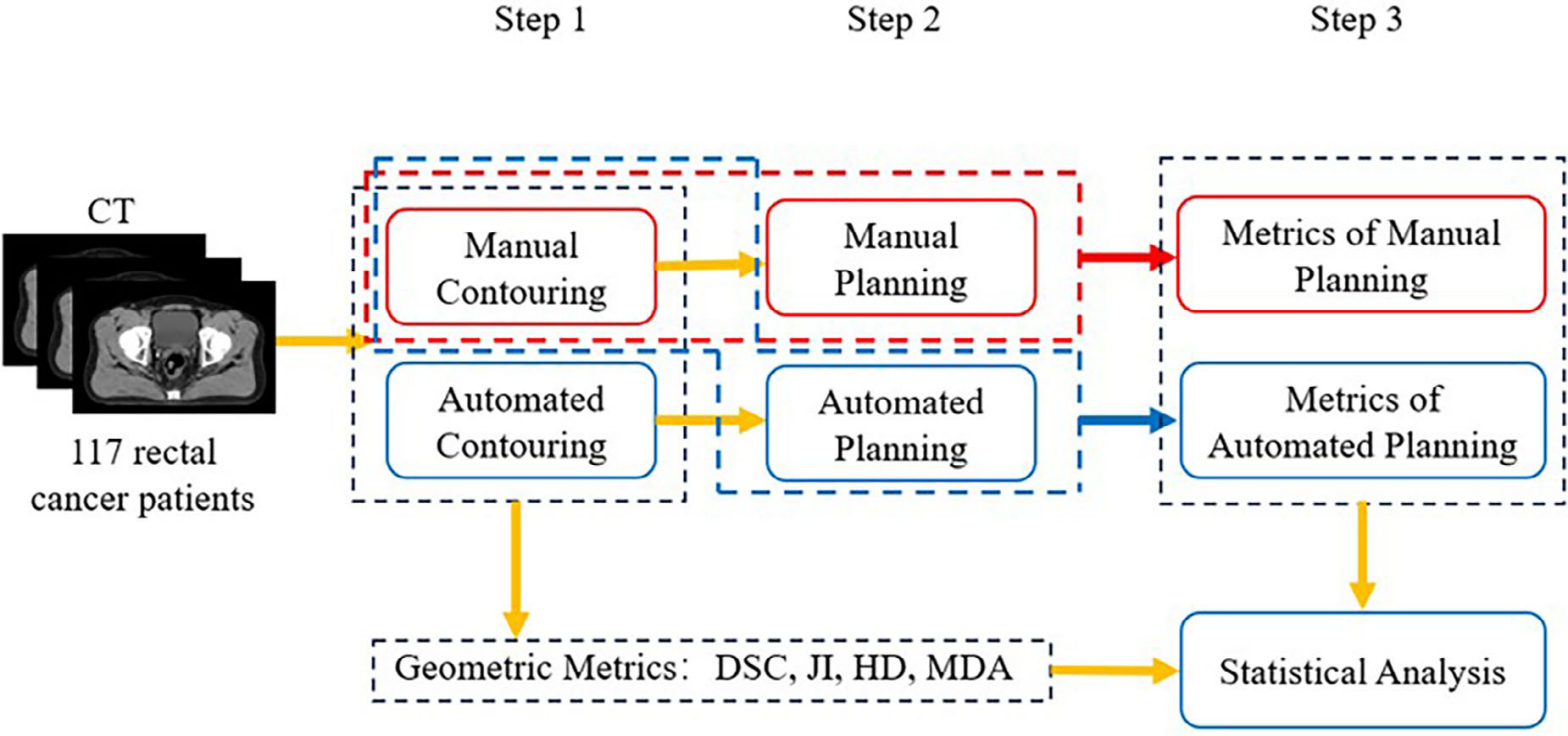
Workflow of this study.

The workflow of this study is illustrated in Figure 2. In Step 1, we select 117 rectal cancer patients who have undergone AIO treatment as the subjects. Next, in Step 2, we re‐contour the ROIs using the AIO process without any manual intervention. Automated planning follows, based on these contoured ROIs. This step ensures that the fields, optimization parameters, and constraints of the new plans remain consistent with those of the manual plans. In Step 3, we incorporate manual contouring into the automated plans to generate two sets of geometric and dosimetric metrics. Finally, we will conduct statistical analyses to draw relevant conclusions. The subsequent sections will elaborate on these procedures.

### 2.1 Patient data

This study involved 117 rectal cancer patients who received AIO treatment between 2020 and 2024. To maintain scientific rigor and reliability, we adhered strictly to the regulations set forth by the ethics committee and obtained informed consent from each participant. Details regarding age, sex, and prescriptions can be found in Table 1.

**TABLE 1:** Clinical characteristics of patients.

Characteristics	Number of patients (*n* = 117)
Age (years)	
19–39	10 (8.5%)
40–59	55 (47.0%)
>60	52 (44.4%)
Gender	
Male	72 (61.5%)
Female	45 (38.4%)
Prescription	
25 Gy/5F	77 (65.8%)
50 Gy/25F	36 (30.8%)
Others	4 (3.4%)
Technology	
VMAT	116 (99.1%)
IMRT	1 (0.9%)

### 2.2 ROI contouring and treatment planning

The “All‐in‐one” workflow consolidates key multiple RT steps, including simulation, contouring, planning, image guidance, beam delivery, and patient‐specific in vivo quality assurance (QA). It is implemented on the newly commercialized CT‐integrated linear accelerator, the uRT‐linac 506c (United Imaging Healthcare, UIH, Shanghai, China). This device features a 16‐slice helical CT scanner coupled with a C‐arm linac. The onboard CT scanner has a bore diameter of 70 cm and supports two imaging modes: diagnostic quality (CTDI dose: 10–50 mGy) for simulation and low dose (CTDI dose: 3.5–5 mGy) for image guidance. The linac delivers a 6‐MV photon beam. Control of this hybrid system is managed by proprietary software, which includes both a treatment planning and oncology information system (uRT‐TPOIS) and a treatment delivery system (TDS), utilizing a shared patient database. Further details about the uRT‐linac 506c platform can be found in a previous report.^14^

Within this workflow, AI modules in the uRT‐TPOIS—developed collaboratively by our department and UIH—perform essential tasks such as contouring and planning. Automated segmentation of organs and target volumes is achieved through a multiresolution VB‐Net convolutional neural network (CNN), utilizing a cascade coarse‐to‐fine strategy to expedite the contouring process.^15^, ^16^ For automated planning, we employ a hybrid voxel‐based optimization approach that integrates dose distribution predictions from a U‐Net network with established clinical objectives, including target homogeneity, conformity, management of cold/hot spots, and dose‐volume constraints for organs at risk.^17^, ^18^ These deep learning models were constructed and trained using our clinical data, and their implementation into the uRT‐TPOIS involved necessary interfaces for clinical application.

Following the completion of the CT scans, the processing and analysis steps occurred as follows: First, the AIO automated contouring algorithm provided preliminary delineation of the tumor region, primarily including the PTV and surrounding OARs such as the bladder, femoral heads, and small bowels. An experienced oncologist subsequently reviewed and adjusted the automated contours to ensure their accuracy and completeness. An automated treatment plan was then generated for the target area and subsequently reviewed and modified by an experienced medical physicist to confirm that the dose parameters met clinical requirements. This plan is referred to as the manual plan.

In this retrospective analysis study, we applied the AIO automated contouring algorithm to regenerate the ROIs, and an automated treatment plan was created based on these new ROIs with no manual intervention throughout the entire process. This plan is termed the automated plan. The new plans maintain the same beam fields, optimization parameters, and constraint functions as the manual plans, ensuring consistency and comparability. Table 2 presents the constraints and dosimetric metrics requiring statistical analysis for the two most common prescriptions among the 117 cases (50 Gy/25F, 25 Gy/5F).

**TABLE 2:** ROI constraints and dosimetric metrics of prescription.

ROI	Constraints		Dosimetric metrics
	Prescription 50 Gy/25F	Prescription 25 Gy/5F	
PTV	Prescription = 50 Gy D_max _< 52.5 Gy	Prescription = 25 Gy D_max _< 26.5 Gy	D_mean_、D_max_、D_min_、D_95_、HI、CI
Bladder	D_mean _< 40 Gy	D_mean _< 12 Gy	D_mean_
Femoral heads	D_mean _< 18 Gy	D_mean _< 9 Gy	D_mean_
Small bowels	/	/	D_max_

### 2.3 Geometric metrics

The effectiveness of both automated and manual contouring was assessed using the Dice similarity coefficient (DSC), Jaccard index (JI), Hausdorff distance (HD), and mean deviation area (MDA). The DSC quantifies the similarity between two sets by calculating the ratio of their intersection to their union. If we let X and Y represent the two regions, then DSC is defined as follows:

$$DSC=\frac{2\left|X\cap Y\right|}{\left|X\right|+\left|Y\right|}$$

The DSC ranges from 0 to 1; a higher value indicates a greater degree of overlap between the two regions, making DSC an essential standard for evaluating segmentation algorithm performance in medical image segmentation.^19^, ^20^ The JI quantifies the similarity between two regions by calculating the ratio of their intersection to their union. Its calculation formula is:

$$JI=\frac{\left|X\cap Y\right|}{\left|X\cap Y\right|}$$

The JI ranges from 0 to 1; higher values indicate greater similarity. This index is commonly employed in medical image processing to evaluate the segmentation performance of tumor regions.^21^, ^22^ In contrast, the HD calculates the maximum distance between two sets of points, representing the worst‐case similarity in shape between the regions. The formula for this calculation is:

$$HD=\max\left(sup_{x\in X}inf_{y\in Y}d\left(x,y\right),sup_{y\in Y}inf_{x\in X}d\left(y,x\right)\right)$$

Here, d(x,y) represents the distance between points x and y, sup denotes the supremum, and inf denotes the infimum. The HD is effective in capturing extreme differences in boundary shapes and is important for assessing structural similarity between those shapes.^23^, ^24^ In contrast, the MDA measures the average distance between multiple pairs of corresponding points, indicating the consistency between two regions. The calculation formula is:

$$MDA=\frac{1}{n}\;\sum_{i=1}^{n}d\left(x_{i},y_{i}\right)$$

Here, n is the number of point pairs, $x_{i}$ and $y_{i}$ are corresponding points on the boundaries of the two regions, and $d(x_{i},y_{i})$ represents the distance between points $x_{i}$ and $y_{i}$. The MDA offers a comprehensive approach to assessing the similarity between two regions. It effectively reduces the influence of single‐point errors on the results.^25^

### 2.4 Dosimetric metrics

Statistical analyses were conducted to compare the dosimetric metrics of automated and manual planning techniques. For the PTV, the key metrics evaluated include the mean dose (D_mean_), maximum dose (D_max_), minimum dose (D_min_), 95% dose volume (D_95_), homogeneity index (HI),^26^ and conformity index (CI).^27^ In evaluating the OARs, the primary dosimetric metrics are the mean dose for the bladder and femoral heads, as well as the maximum dose for the small bowel. All statistical dosimetric metrics are summarized in Table 1. The calculations for HI and CI are provided below:

$$\mathrm{HI}=\frac{D_{2}-D_{98}}{D_{50}}$$

$$\mathrm{CI}=\frac{V_{ri}}{V_{ptv}}$$

Here, $D_{x}$ is the dose corresponding to the target volume of x%, $V_{ri}$ is the absolute volume that reaches the prescription dose, and $V_{ptv}$ is the absolute volume of the PTV.

### 2.5 Statistical analysis

This study utilized several statistical analysis methods: Spearman correlation analysis, two‐tailed paired *t*‐tests, and the IQR method. Spearman correlation was chosen because our Shapiro‐Wilk tests revealed non‐normal distributions in the geometric metrics (*p* < 0.05), making parametric methods like Pearson correlation inappropriate. Additionally, preliminary data exploration showed nonlinear relationships between variables that Spearman can better capture through its rank‐based approach, while also being less sensitive to outliers in our dataset. This method provides robust correlation estimates without requiring assumptions about linearity or data distribution. Prior to performing the paired *t*‐tests, the normality of the data was assessed using the Shapiro‐Wilk test. To ensure accuracy and reliability, all statistical analyses were performed using SPSS 26.0 software. We established a significance level of 0.05 for all tests to assess the statistical significance of the results.

## 3 RESULTS

### 3.1 Distribution of geometric metrics

**FIGURE 3:**
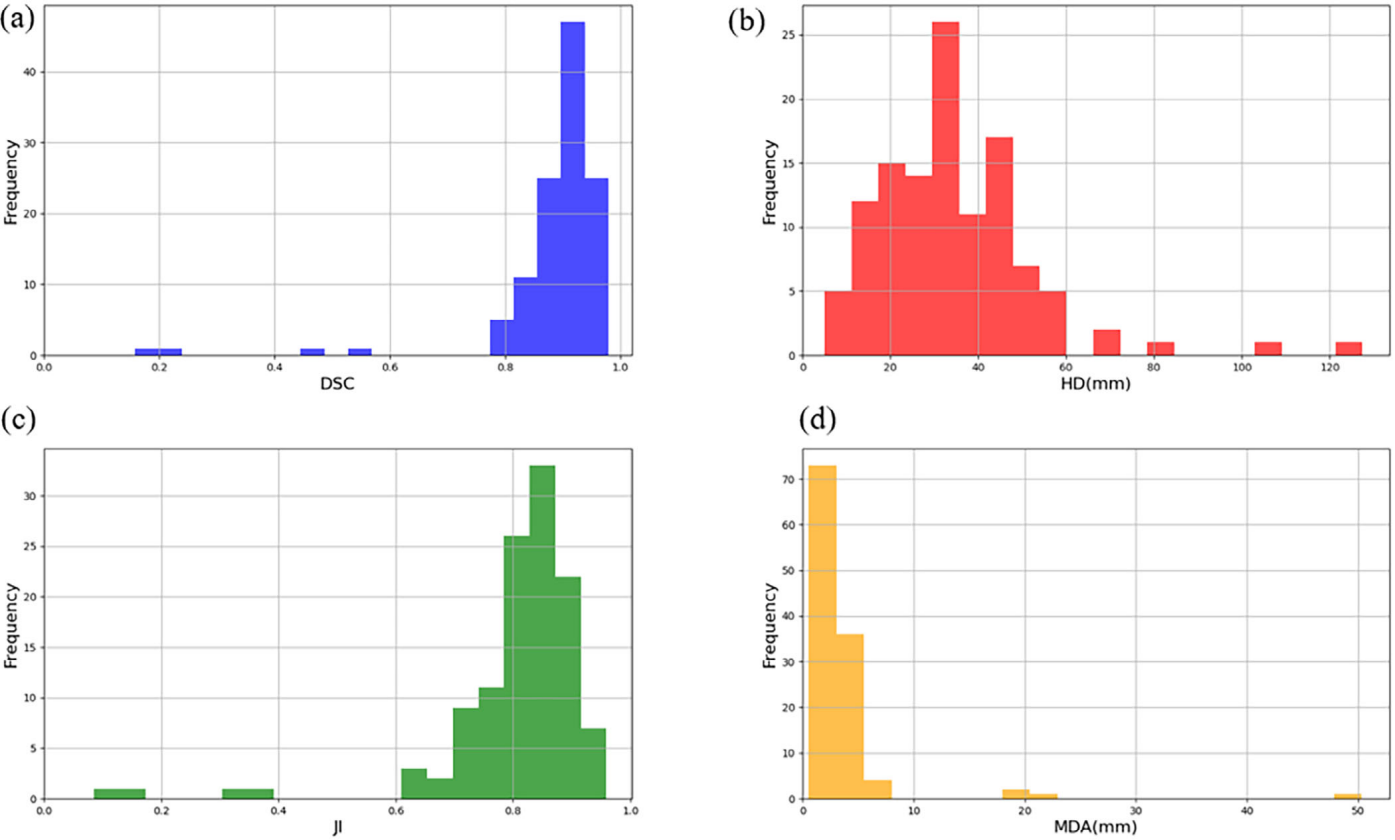
Bar charts of distribution of DSC (a), JI (b), HD (c), and MDA (d).

Figure 3 presents the distribution of geometric evaluation metrics—DSC, JI, HD, and MDA—comparing automated contouring with manual contouring. The automated contouring algorithm effectively delineates the PTV in most cases, achieving a mean DSC greater than 0.9 and a mean JI exceeding 0.8. These values are comparable to those reported for manual contouring in previous studies, where a DSC above 0.9 are regarded as excellent.^28^ This suggests that the automated algorithm performs at a level consistent with or exceeding manual contouring in terms of geometric accuracy.

### 3.2 Correlation between dosimetric metrics and geometric metrics

**FIGURE 4:**
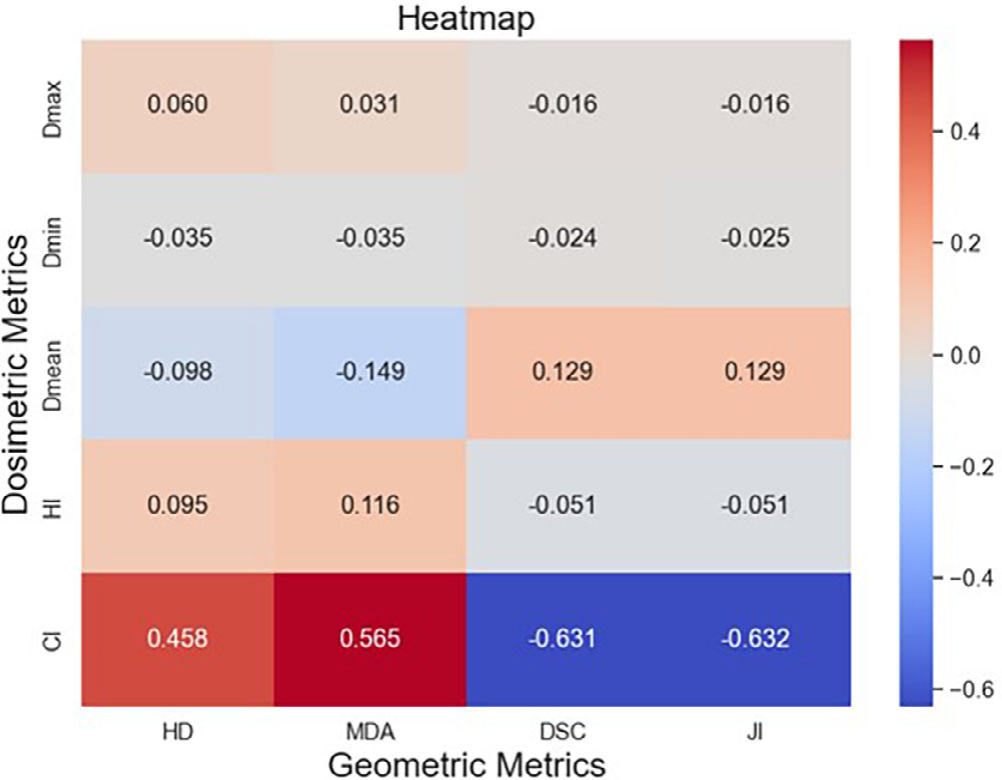
Heatmap of geometric and dosimetric metrics.

Figure 4 presents a heatmap derived from the Spearman correlation coefficient matrix, illustrating the relationships between PTV dosimetric metrics and geometric metrics. The results indicate that ΔCI revealed moderate correlations with all four geometric metrics: HD (|ρ| = 0.458, *p* < 0.01), MDA (|ρ| = 0.565, *p* < 0.01), DSC (|ρ| = 0.631, *p* < 0.01), and JI (|ρ| = 0.632, *p* < 0.01). These moderate correlations suggest that while geometric metrics, such as target volume overlap and boundary accuracy, may influence dose conformity, they are not the sole determinants of treatment plan quality. These findings suggest that geometric metrics should be integrated with dosimetric and patient‐specific factors for more comprehensive treatment evaluation. In contrast, other dosimetric metrics exhibited no significant correlations with the geometric metrics (|ρ| < 0.2), indicating their limited role in predicting dose distribution based on geometric parameters alone.

### 3.3 Dosimetric metrics of OARs

**FIGURE 5:**
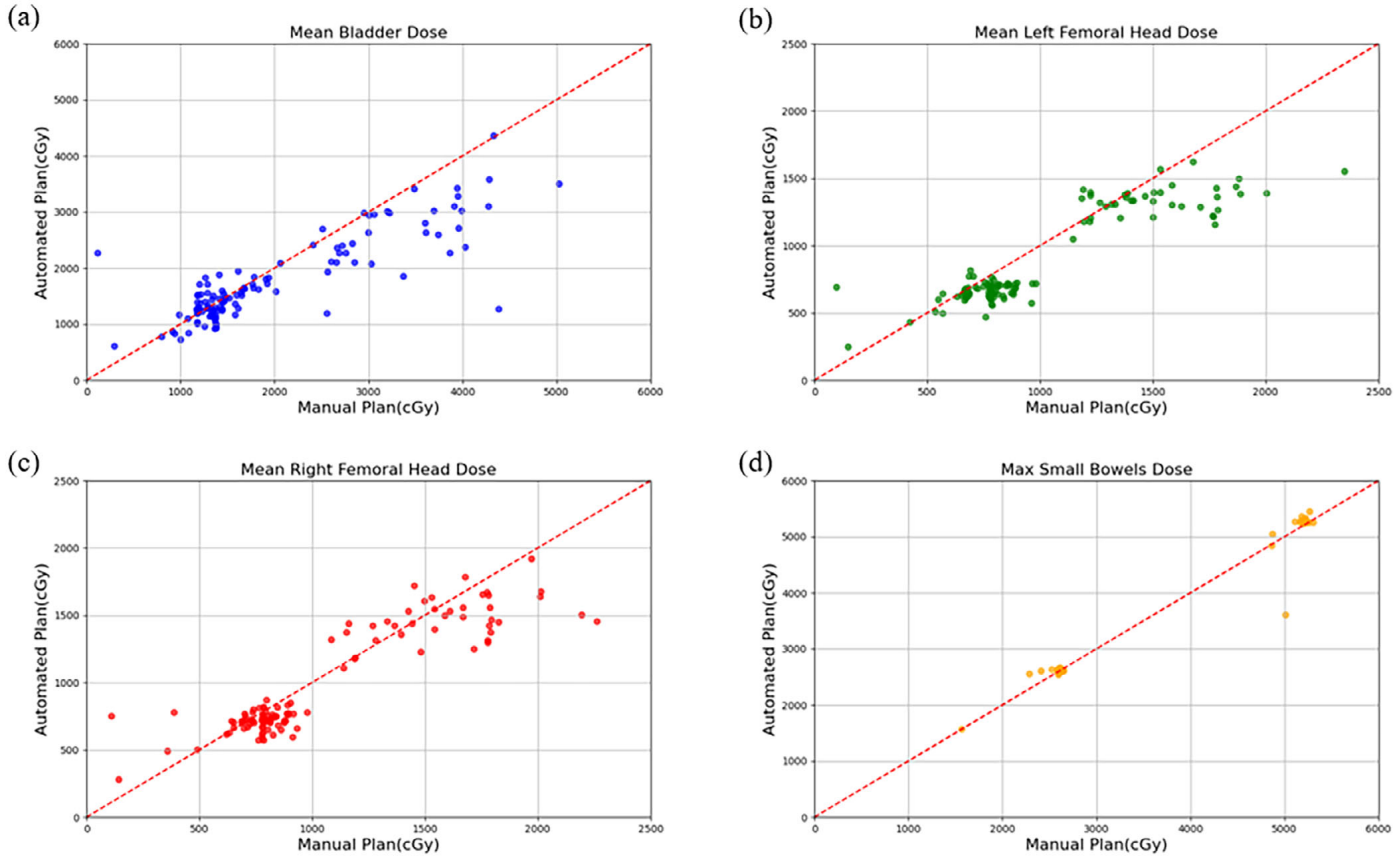
Scatter plot of dosimetric metrics [mean bladder dose (a), mean dose to left femoral head (b), mean dose to right femoral head (c), and maximum dose to the small bowel (d)] for manual and automated plans.

**FIGURE 6:**
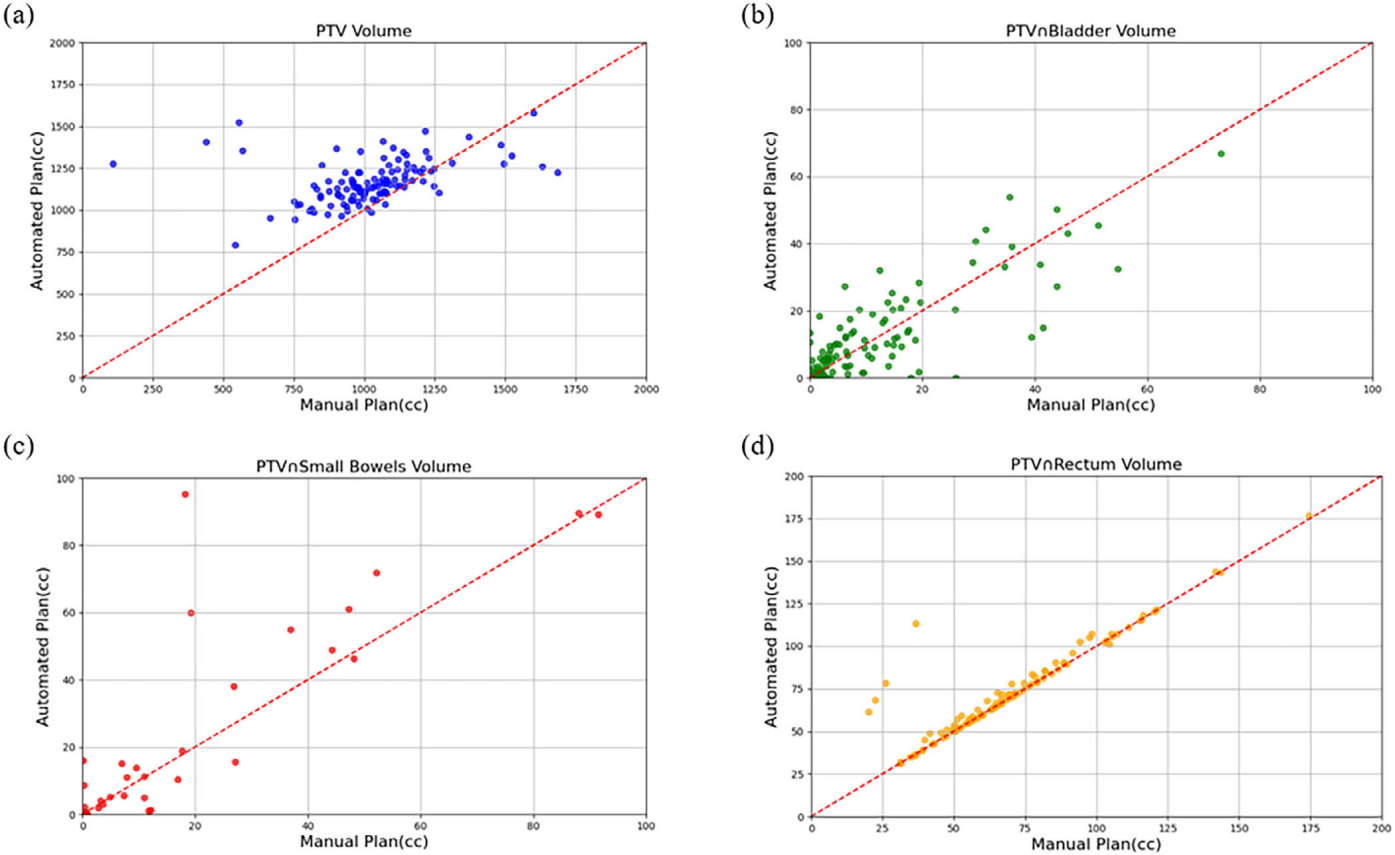
Scatter plot of geometric metrics [PTV volume (a), PTV ∩ bladder volume (b), PTV ∩ small bowels volume (c), and PTV ∩ rectum volume (d)] for manual and automated plans.

The study conducted a paired *t*‐test on key dosimetric metrics for OARs and generated a scatter plot (Figure 5). Automated plans significantly reduced the mean dose to the bladder (*p* < 0.01) and femoral heads (*p* < 0.01) when compared to manual plans. However, there was no significant difference in the maximum dose to the small bowel (*p* = 0.44). Additionally, we performed a paired t‐test on relevant geometric metrics, including volume PTV, PTV ∩ bladder, PTV ∩ small bowel, and PTV ∩ rectum (Figure 6). To facilitate a more direct comparison of the dosimetric parameters between AIO and manual plans, we have included a summary table (Table 3) that presents the key metrics for both approaches. The results showed that the PTV volume delineated by automated contouring was significantly larger than that from manual contouring (*p* < 0.01). Regarding the OARs, the overlapping volume of the rectum with the PTV displayed a decreasing trend in automated planning compared to manual planning (*p* < 0.01), while no significant differences were noted in the overlapping volumes of the bladder and small bowel with the PTV.

**TABLE 3:** Key dosimetric metrics of OARs.

		Prescription 50 Gy/25F		Prescription 25 Gy/5F	
Organs at risk	Metrics (Gy)	Automated plans	Manual plans	Automated plans	Manual plans
Bladder	Mean dose	27.0 ± 6.8	33.5 ± 7.5	14.0 ± 3.2	14.2 ± 3.3
Left femoral head	Mean dose	13.4 ± 1.6	15.5 ± 2.4	6.6 ± 1.1	7.8 ± 1.5
Right femoral head	Mean dose	14.7 ± 2.0	16.1 ± 2.4	7.3 ± 1.5	7.8 ± 1.6
Small bowel	Max dose	52.5 ± 1.3	52.0 ± 1.2	26.1 ± 0.4	26.0 ± 0.5

All values represent mean ± 1 standard deviation (SD) unless otherwise noted.

### 3.4 IQR method analysis of the key metrics for plan quality

**FIGURE 7:**
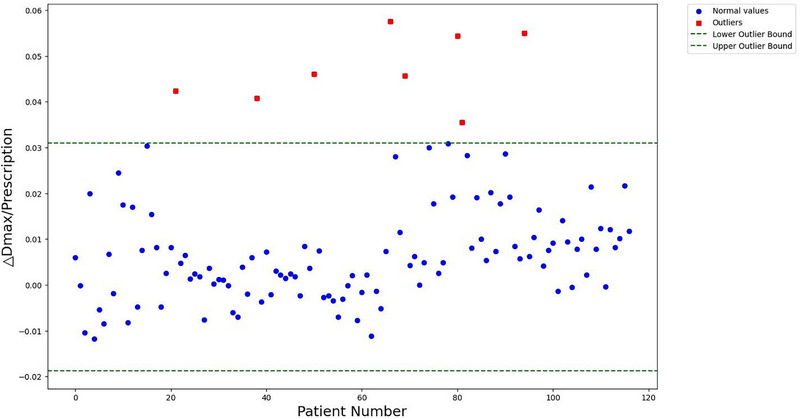
IQR scatter plot with outlier identification of △D_max_/Prescription.

**FIGURE 8:**
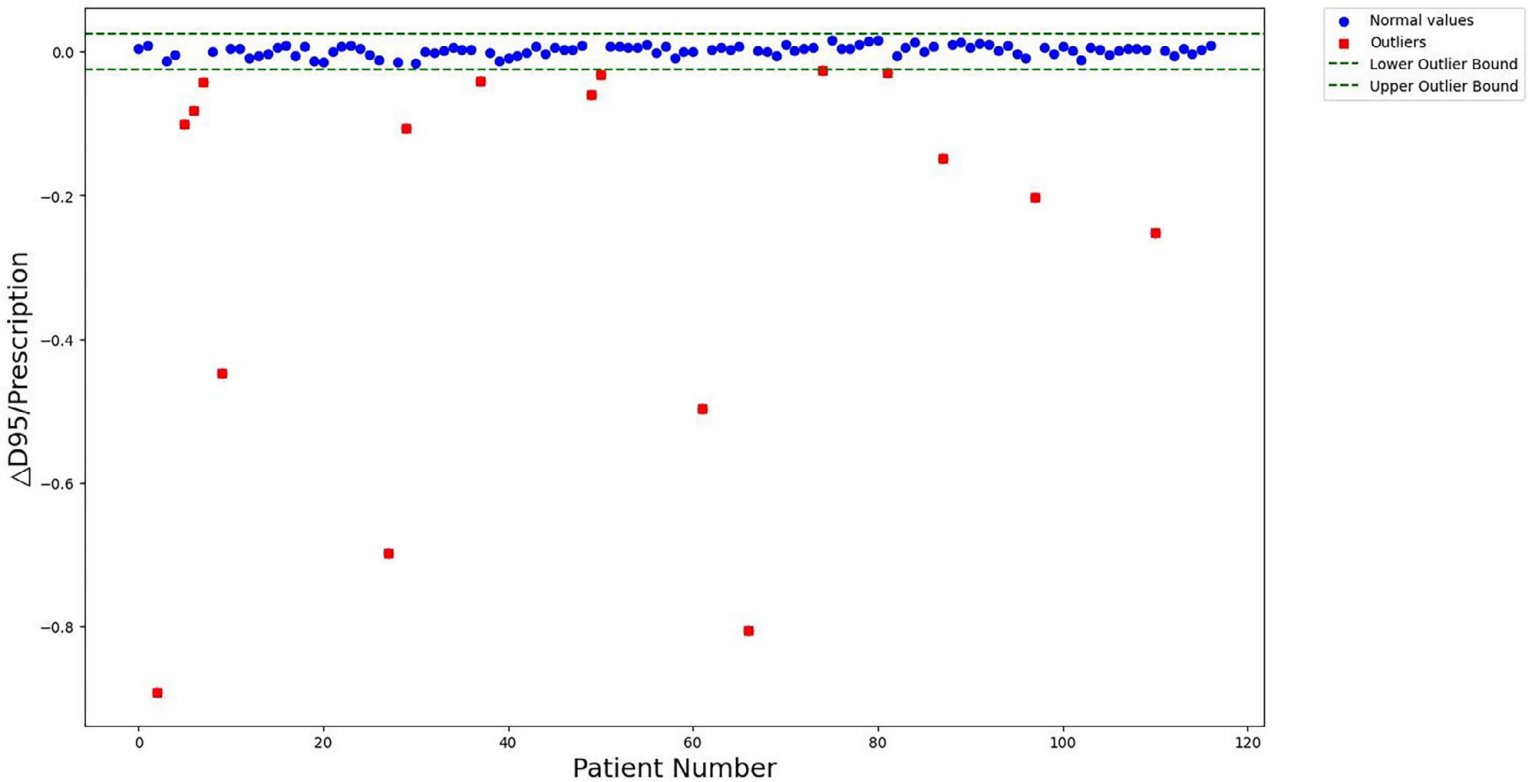
IQR scatter plot with outlier identification of △D_95_/Prescription.

To determine whether a plan meets clinical standards, it is essential to evaluate several metrics, with the D_max_ and the D_95_ being the most important. In this study, we conducted an IQR analysis on these dosimetric metrics (Figures 7 and 8), identifying outliers as plans that fail to meet clinical requirements. The figures illustrate the distribution of variations in D_max_ and D_95_ for both automated and manual plans, highlighting outliers in red. The outliers were defined as values falling outside Q1‐1.5×IQR or Q3+1.5×IQR. These outlier plans were retained in the overall analysis to provide a conservative estimate of clinical acceptability, but were specifically flagged for clinical review as they represented cases requiring manual intervention (*n* = 22/117). After excluding these outliers, the proportion of clinically acceptable plans within the automated AIO workflow is 81.2% (95 out of 117).

## 4 DISCUSSION

Accurate target delineation is crucial in RT, significantly influencing treatment outcomes. Recent research on geometric metrics for target areas has created a robust framework and identified key parameters that effectively reflect spatial similarity. For example, Sun et al. demonstrated the power of deep learning in brain tumor segmentation, achieving a DSC of 0.92 and a JI of 0.86, which indicates excellent segmentation performance.^28^ Additionally, HD is an important metric for assessing the accuracy of segmentation boundaries. Weng et al. reported that their enhanced segmentation model significantly lowered HD values in lung cancer target contouring, thus improving the precision of tumor margins.^29^ The MDA metric also helps clinicians intuitively evaluate segmentation quality. For instance, Urago et al. effectively used MDA to assess the deviations between automated segmentation and manual delineation in prostate cancer patients.^30^ Together, these geometric metrics serve as essential references for delineating tumor target volumes, highlighting their critical role in optimizing RT treatment plans.

While geometric metrics assess tumor region overlap, they do not fully capture dose distribution, limiting their predictive value. As a result, there has been an increasing reliance on dosimetric metrics in recent years. Key metrics such as the HI and CI play an essential role in evaluating the effectiveness of dose delivery to tumor targets, all while safeguarding OARs.^31^, ^32^, ^33^ Moreover, these dosimetric metrics not only act as vital references for assessing plan quality but also pave the way for enhancing automated treatment planning.

Based on these observations, the feasibility and clinical pass rates of automated RT plans have been assessed using multiple indicators. Cilla et al. found that treatment plans generated by automated algorithms resulted in superior target dose conformity and homogeneity compared to other tangential techniques.^34^ Additionally, He et al. found that automated plans for liver cancer stereotactic body RT provided similar planning target volume dose coverage and statistically superior organ at risk sparing compared to manual plans.^35^ Furthermore, Krayenbuehl et al. demonstrated the benefits of automated technologies in improving treatment consistency and minimizing the risk of side effects in their research on brain cancer.^36^ Finally, Gao et al. compared automated planning with conventional planning techniques on prostate cancer, showing that automated planning provided a substantial advantage in treatment planning, achieving a score over 142.1/150.^37^

However, the clinical adoption of these automated planning methods varies. The automatic treatment planning system (aTPS) for whole‐brain radiotherapy (WBRT) has shown promising results in achieving dose constraints and improving plan quality, but its clinical implementation is still limited to specific centers.^36^ The virtual treatment planner (VTP) for prostate stereotactic body radiation therapy (SBRT) has demonstrated high performance in generating clinically acceptable plans, but its reliance on deep learning models and the need for extensive training data may pose challenges for broader adoption.^37^ In contrast, the AIO technology for rectal cancer RT offers a more comprehensive solution by integrating multiple steps into a single workflow, potentially reducing the barriers to clinical adoption.

Despite these advancements, limitations remain. The AIO technology, like other automated methods, may struggle with small or asymmetrical target volumes, requiring manual intervention to ensure optimal treatment outcomes.^34^ Additionally, the reliance on high‐quality training data and the need for continuous validation studies may limit its widespread acceptance among radiation oncologists. Future work should focus on improving the adaptability of AIO systems to handle more complex cases and enhancing their clinical validation to address these limitations.

**FIGURE 9:**
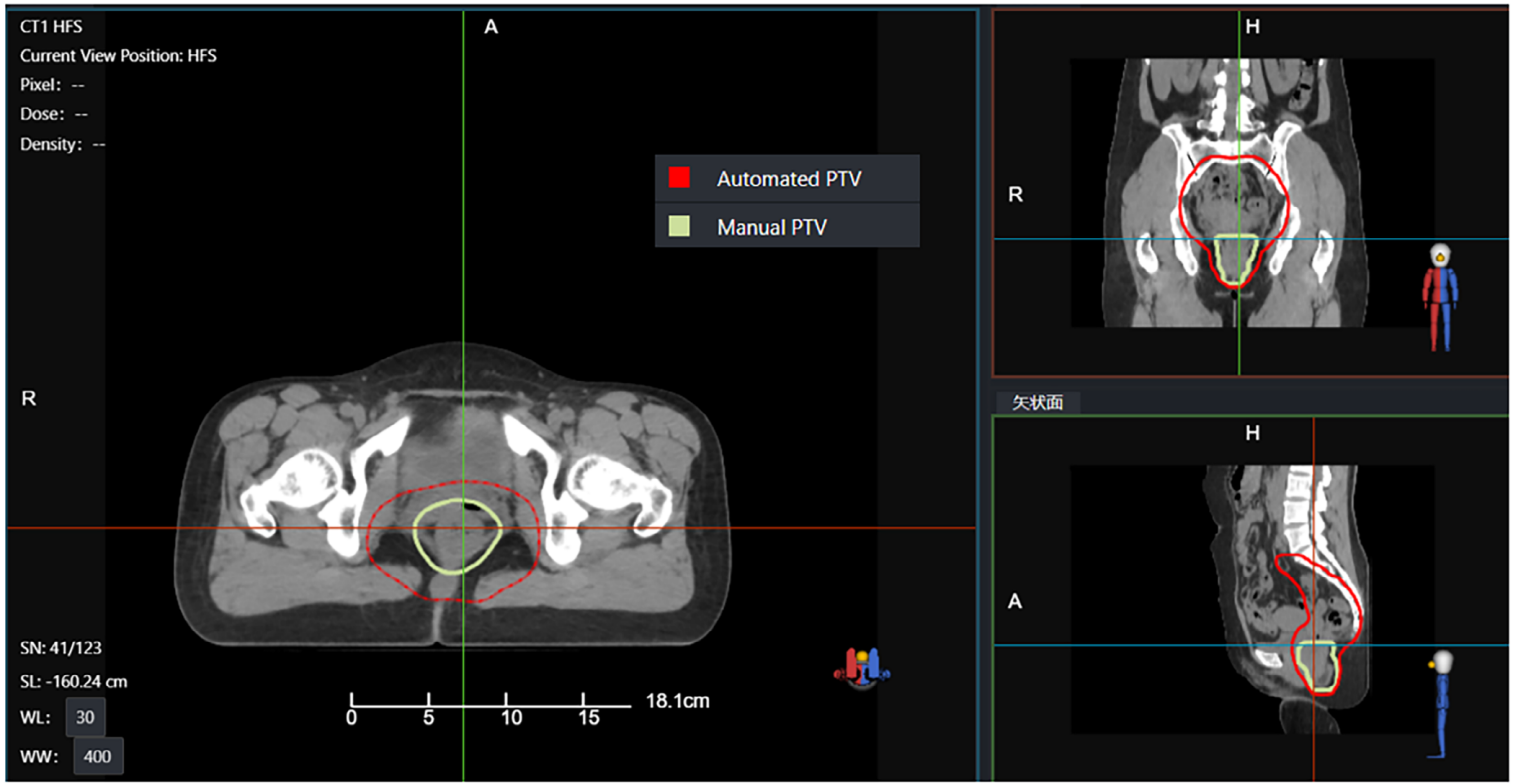
PTV contouring of patient A.

**FIGURE 10:**
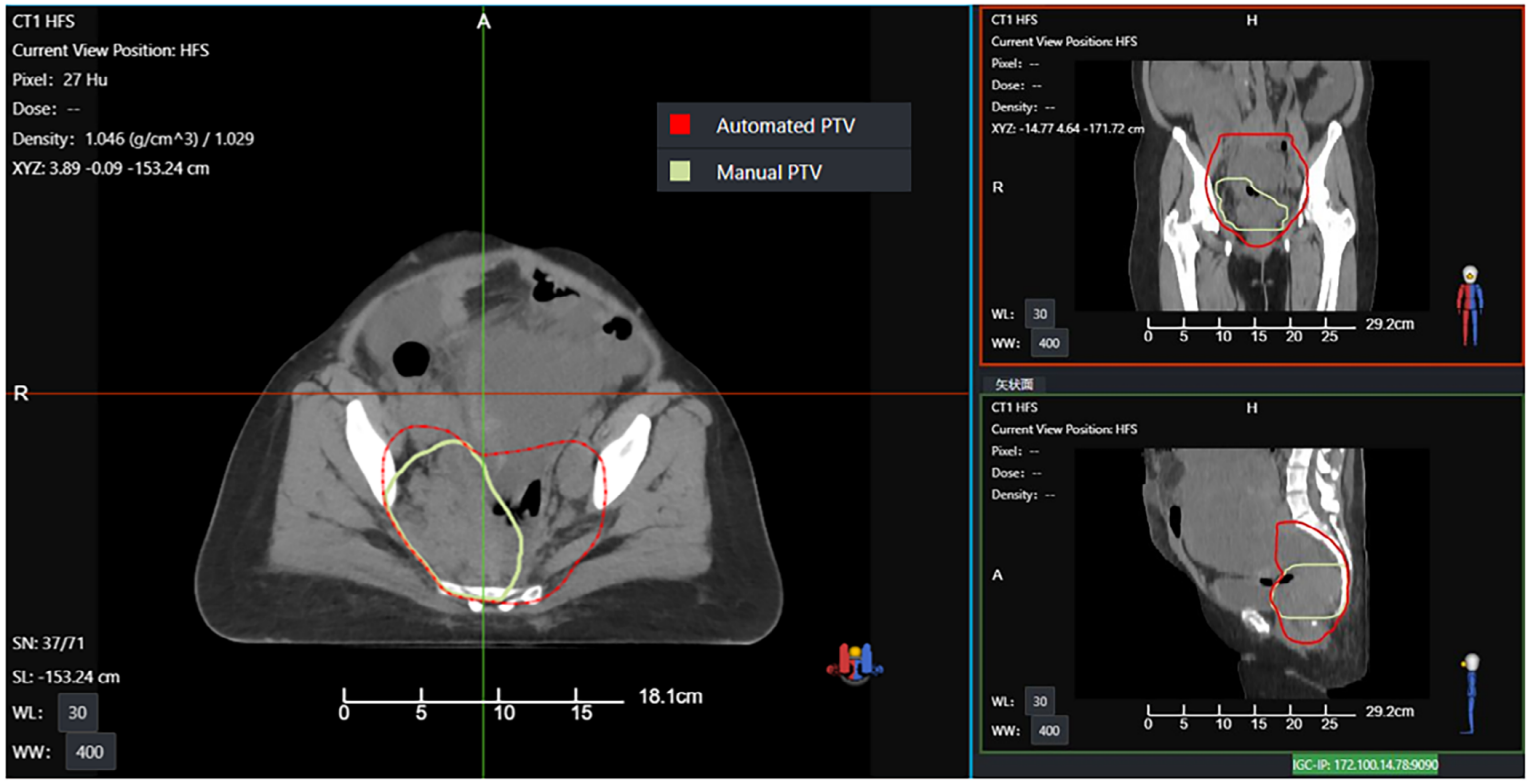
PTV contouring of patient B.

**FIGURE 11:**
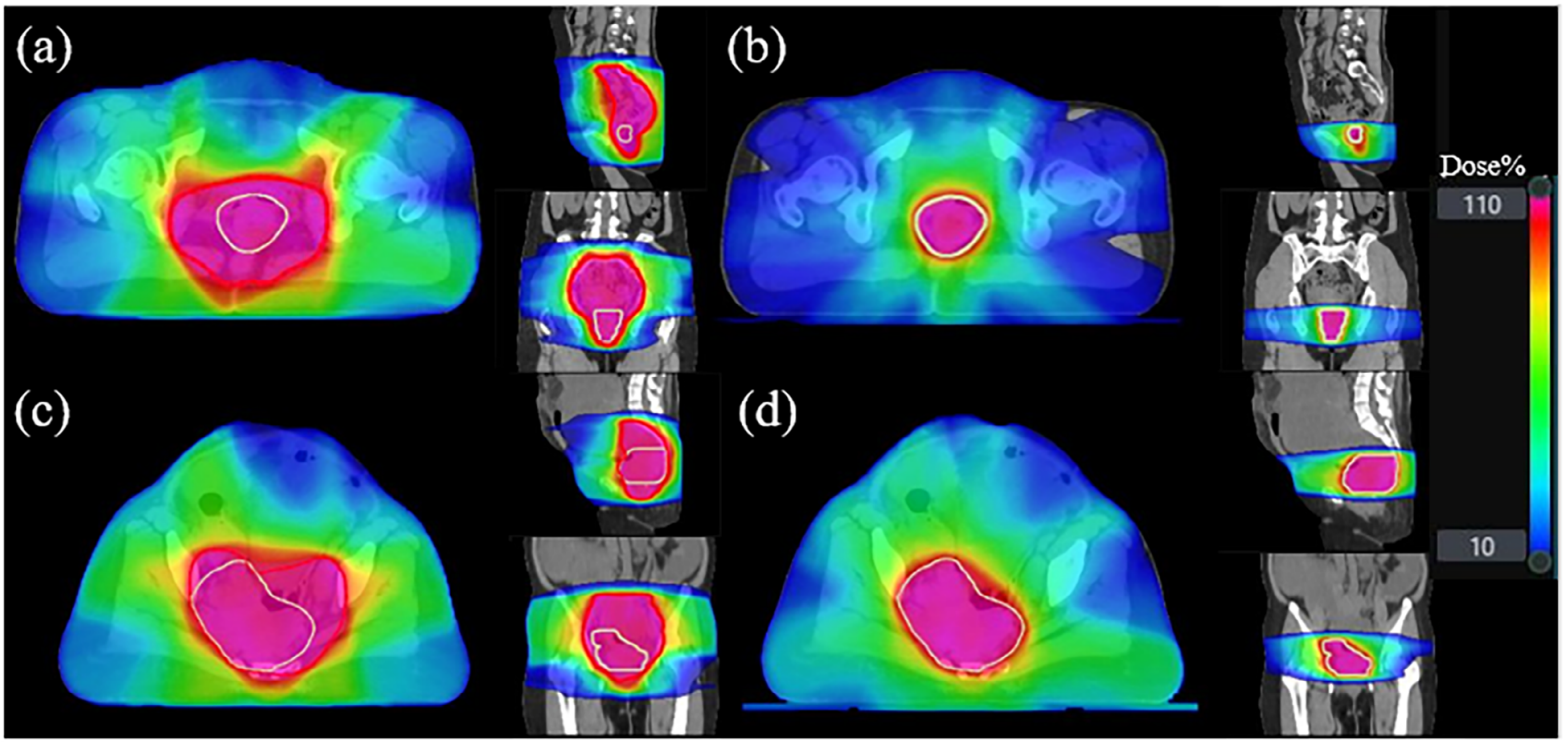
Dose distributions [Patient A automated plan (a), Patient A manual plan (b), Patient B automated plan (c), and Patient B manual plan (d)].

**FIGURE 12:**
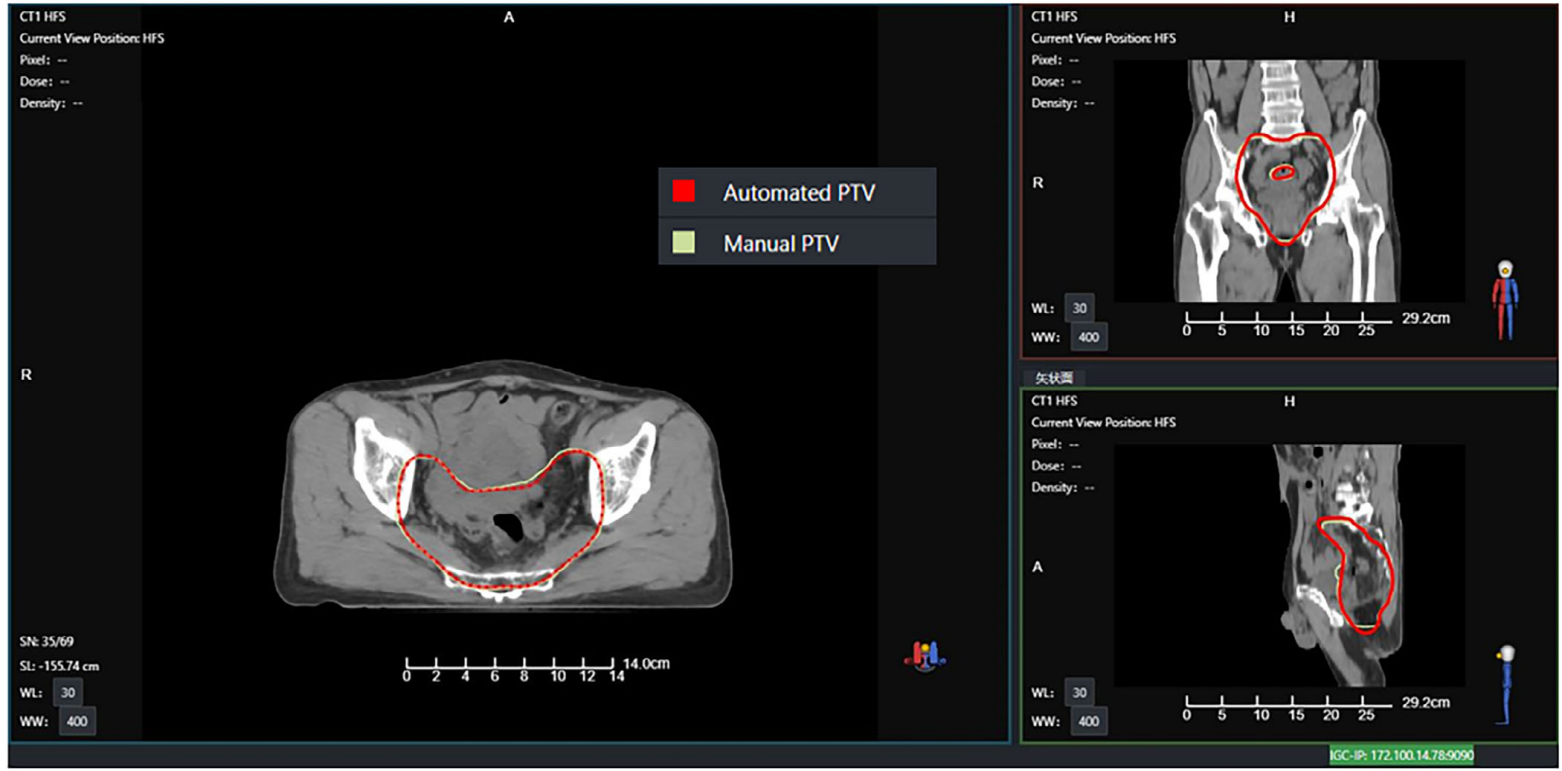
PTV contouring of patient C.

In this study, we conducted a detailed analysis of several plans that did not meet clinical requirements. Figures 9 and 10 present CT images of two patients (patient A and B), along with the PTVs generated during two contouring sessions. Figure 11 shows the dose distributions of the manual and automated plans for these two patients. These PTVs show significant deviations from the standard contours. Figure 12 provides an example (patient C) of a PTV that meets the standard. In these images, red denotes the automatically generated PTV, while green signifies the manually modified PTV. The figures show that the upper and lower boundaries of the PTV target areas in these cases experienced significant changes following physician modifications, whereas such changes were not prominently reflected in the AIO automatically contoured target areas. Based on the earlier statistical results, we hypothesize that the absolute volume of the PTV affects the efficacy of AIO treatment. Specifically, when the length of the PTV in the head‐to‐foot direction is considerably smaller than that of conventional rectal tumor target areas, AIO's performance diminishes. Additionally, in the second case, the manually contoured PTV displayed notable geometric asymmetry in the left‐right direction, unlike the AIO automatically contoured PTV, which showed minimal asymmetry. Thus, we can posit that AIO may lack the necessary sensitivity to left‐right asymmetrical rectal tumor target areas.

Future research should focus on improving AIO algorithms to better handle small or asymmetrical target volumes. For example, incorporating advanced deep learning models trained on a wider variety of tumor geometries could enhance the system's ability to adapt to complex cases. Additionally, integrating real‐time feedback from clinicians during the contouring process could help bridge the gap between automated and manual planning, further improving the reliability and acceptance of AIO technology in clinical practice. Adaptive planning features, such as dynamic adjustment of optimization parameters based on real‐time imaging data, could also be explored to enhance the precision and flexibility of AIO systems. Machine learning techniques, such as reinforcement learning, could be employed to fine‐tune segmentation and planning algorithms, potentially improving the accuracy of target delineation and dose distribution.

In conclusion, during clinical treatment, timely manual intervention is critical when the head‐to‐foot length of the rectal tumor target area is significantly small or demonstrates pronounced asymmetry, providing practical guidance for implementing AIO in clinical settings.

The dosimetric metrics of OARs reveal that automated treatment plans significantly reduce the average dose to both the bladder and femoral heads. This reduction is clinically significant, as lower doses to the bladder are associated with reduced risks of acute and chronic urinary toxicity,^38^ while lower doses to the femoral heads may decrease the likelihood of radiation‐induced osteonecrosis.^39^ This improvement likely stems from AIO technology's emphasis on safeguarding OARs through precise contouring and optimized dosing algorithms, highlighting its potential to enhance both short‐term and long‐term patient outcomes.

Interestingly, clinicians often opt for contractions instead of expansions when delineating targets defined by AIO. This tendency may be related to the differences between automated contouring and clinicians' understanding of the expansion from (gross target volume) GTV to PTV. In future studies, we will focus more on the automatic contouring of PTV, aiming to provide a more comprehensive explanation for this phenomenon. Contraction operations are notably more common in the rectal regions. This trend may stem from their commitment to maintaining the patient's quality of life post‐RT, as reducing the target volume near critical structures such as the rectum can minimize the risk of toxicity.

## 5 CONCLUSION

Analysis indicates that in routine clinical practice, AIO treatment plans achieved clinical treatment standards without manual intervention in approximately 81.2% of cases. In addition, the dosimetric metrics for some OARs in the AIO treatment plans have also significantly decreased, indicating better sparing of these critical structures and potentially reducing the risk of toxicity. These findings highlight the efficiency and safety of AIO treatment plans, supporting their use as a reliable tool in clinical practice. Furthermore, oncologists typically preferred to use contractions rather than expansions when modifying the auto‐contouring of the PTV, particularly at the boundary between the PTV and the rectal area. This preference may reflect a cautious approach to safeguarding patient quality of life post‐RT.

In practical terms, these results suggest that AIO technology can significantly streamline RT workflows, particularly in high‐volume clinical settings, while maintaining or even improving treatment quality. However, the need for manual intervention in certain cases, such as small or asymmetrical target volumes, underscores the importance of integrating AIO with clinician expertise. Future implementations of AIO should focus on enhancing its adaptability to complex cases and improving its acceptance among radiation oncologists through and robust validation studies.

## AUTHOR CONTRIBUTIONS

Haoyang Zhai: Writing—original draft, Conceptualization, Data curation, Formal analysis, Investigation, Validation. Jiazhou Wang: Writing—review & editing, Methodology, Resources, Supervision. Weigang Hu: Writing—review & editing, Resources, Supervision.

## CONFLICT OF INTEREST STATEMENT

The authors declare no conflicts of interest.

## ETHICS STATEMENT

This study involved 117 rectal cancer patients of Fudan University Shanghai Cancer Center between 2020 and 2024. To maintain scientific rigor and reliability, we adhered strictly to the regulations set forth by the ethics committee and obtained informed consent from each participant.
